# Research on Clustering Algorithm Based on Improved SOM Neural Network

**DOI:** 10.1155/2022/1482250

**Published:** 2022-08-10

**Authors:** Chengxiang Shi, Xiaoqing Li

**Affiliations:** Department of Mathematics and Information Engineering, Chongqing University of Education, Chongqing, China

## Abstract

Clustering algorithm is a statistical method to study sample classification. With the rapid development of science and technology, people have higher and higher requirements for data classification, so there are more and more researches on clustering in modern society. Various mathematical algorithms are introduced to further improve the accuracy of clustering. Therefore, this paper proposes an improved SOM neural network algorithm to evaluate the comprehensive quality of students. SOM neural network can automatically find the internal laws and essential attributes in the samples, self-organize and adaptively change the network parameters and structure, and realize the classification of samples. Factor analysis is introduced to reduce the dimension of input layer in SOM neural network analysis, better process high-dimensional data, and improve the speed and accuracy of the algorithm. The improved SOM neural network algorithm can be used for the cluster analysis of the comprehensive quality of college students. The algorithm simulation results show that the improved neural network algorithm can intuitively evaluate the comprehensive quality of students and reflect the overall characteristics of each type of student.

## 1. Introduction

With the advent of the era of big data, the sources of data are becoming richer and richer, and the amount of data also shows a trend of rapid growth. Research and mining of important information contained in data have become a specialty. At present, data mining technology is widely used in various fields, such as economy, finance, transportation, commerce, and education. Cluster analysis is also an important task in data mining. It can find out the laws in the data and express them in the form of visualization. At present, there are many applications of data mining in the field of education, such as students' comprehensive quality evaluation. These assessments are also an important basis for students to strengthen learning, teachers to adjust teaching, and schools to arrange courses.

There are many methods for the evaluation of students' comprehensive quality, such as the analytic hierarchy process adopted by Lin [1], the adaptive multiminimum support association algorithm and SOM neural network algorithm of Xie [2], and the SVM method used by Yang et al. [3].

The SOM neural network adopted in this paper is also widely used in practical life. For example, Chen [4] improved the clustering algorithm SOM-K-means to crawl and classify the network water army, which is of great significance to the governance of the network water army. Wu [5] proposed an improved clustering algorithm, SOM-K-medoids-CH, which can effectively and accurately divide a large number of bank customers, mine out their potential needs, and sell the right products to the right customers at the right time.

However, we find that the data for evaluating students are multidimensional, the subject scores are diverse, and the correlation between subjects is relatively complex [6]. Students can be divided into different categories by directly using the clustering method according to the data [7]. However, for researchers, it is difficult to directly observe the commonalities between each type of student from the classification results because of the large and complex data. Moreover, for SOM neural network algorithm, the result is also greatly affected by the input samples [8]. Therefore, in view of the above problems, this paper will introduce factor analysis into the SOM algorithm model to eliminate the relevant influence, extract the important indicators in the data, and analyze and verify the classification results.

## 2. Related Algorithm Theory

### 2.1. Basic Theory of Factor Analysis

Factor analysis was first proposed by British psychologist C. E. Spearman. In his research, he found that there was a certain correlation between students' grades in various subjects and then speculated whether there were some potential common factors affecting students' academic performance. Factor analysis can find out the hidden representative factors in many variables and classify the variables with the same essence into one factor, which can reduce the number of variables and test the hypothesis of the relationship between variables [9–11].

In factor analysis, each factor is not related to each other, and all variables can be expressed as a linear combination of common factors. There *n* are samples *p* and indicators, *X*=(*X*_1_, *X*_2_,…,*X*_*P*_)^*T*^ which are random vectors. If the common factor to *F*=(*F*_1_, *F*_2_,…,*F*_*m*_)^*T*^ be found is, the factor model is

(1) $$\begin{array}{c} X_{1}=a_{11}F_{1}+a_{12}F_{2}+\cdots +a_{1m}F_{m}+\varepsilon_{1},\\ X_{2}=a_{21}F_{1}+a_{22}F_{2}+\cdots +a_{2m}F_{m}+\varepsilon_{2},\\ \ldots \ldots \\ X_{p}=a_{p1}F_{1}+a_{p2}F_{2}+\cdots +a_{pm}F_{m}+\varepsilon_{p}. \end{array}$$

The matrix *A*=(*a*_*ij*_) is called the factor *a*_*ij*_ load matrix, which reflects the *i* importance *X*_*i*_ of *j* the variable *F*_*j*_ to the common factor *ε*. As a special factor, it represents the variation of variables caused by influencing factors other than common factors, which can be ignored in the practical analysis [12, 13].

The model obtained by factor analysis is not affected by dimension, and its factor load is not unique. When the factor load is complex and difficult to be explained reasonably, a new factor load matrix can be obtained by factor rotation, and its analysis significance will be more obvious.

### 2.2. Self-Organizing Mapping Network

Self-organizing feature mapping network was proposed by Professor T. Kohonen of Helsinki University in Finland in 1981, which is called SOM network for short. Kohonen believes that when a neural network receives external input, each region of the neural network will have different response characteristics, and this process is completed automatically.

A typical feature of a feature mapping network is that it can be divided into input layer and competition layer on a one-dimensional or two-dimensional processing unit array. After self-organizing training, neurons will be orderly arranged in the competition layer. Neurons with similar functions are very close, and neurons with different functions are far away.

SOM network adopts the Kohonen algorithm, and the influence of winning neurons on their adjacent neurons is from near to far, from excitation to inhibition. Therefore, not only the winning neurons need to adjust the weight but also the surrounding neurons will adjust the corresponding weight. The learning algorithm steps are as follows:

(1) Network initialization, set the initial value of the weight between the input layer and the mapping layer with a random number.
(2) Normalized data and input data. Normalize the data and input the *x*=(*x*_1_, *x*_2_, *x*_3_,…*x*_*n*_)^*T*^ vector to the input layer.
(3) Calculate the distance between the weight vector of the mapping layer and the input vector. The distance between *j* the second neuron of the mapping layer and the inputvector is
  (2) $$\begin{array}{c} d_{j}=\left\|X-W_{j}\right\|\\ =\sqrt{\sum_{i=1}^{m}{\left(x_{i}\left(t\right)-w_{ij}\left(t\right)\right)}^{2}}. \end{array}$$
where is the weight between *i* the neurons of the input layer *j* and the neurons of the mapping layer.
(4) Define areas of excellence.
(5) Weight learning. The weights of winning neurons and adjacent neurons are updated according to the following formula:
  (3) $$\begin{array}{c} \Delta w_{ij}=\eta h\left(j,j^{*}\right)\left(x_{i}-w_{ij}\right), \end{array}$$
  where *η* is a constant of
  (4) $$\begin{array}{c} h\left(j,j^{*}\right)=\exp\left(-\frac{{\left|j-j^{*}\right|}^{2}}{\sigma^{2}}\right), \end{array}$$
*σ*^2^ decreases with the progress of this learning.
(6) Calculate the *o*_*k*_=*f*(min_*j*_‖*X* − *W*_*ij*_‖) output.
(7) If the requirements are met, output the results, otherwise return to (3) to continue.

## 3. Improved SOM Learning Algorithm

**Figure 1:**
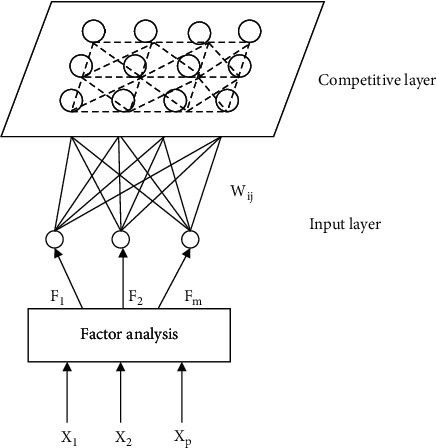
Improved SOM neural network model.

In the improved SOM algorithm, a factor analysis layer is added before the input of SOM sample data. After data are input into factor analysis layer, the factor load matrix table can be obtained by dimensionality reduction of data through factor analysis. By observing the load matrix table, we can get the commonness of each factor after dimensionality reduction and then extract the representative factor and name the representative factor according to the commonness. Then, the extracted data are input into the input layer of the SOM model, and the data are transmitted to the neurons of each competing layer [14, 15]. The improved SOM neural network model is shown in Figure 1.

The first layer is factor analysis. By *n* inputting samples *p* and indicators, *X*=(*X*_1_, *X*_2_,…,*X*_*P*_)^*T*^ the dimensionality of the data is reduced and standardized, the *F*=(*F*_1_, *F*_2_,…,*F*_*m*_)^*T*^ factors are output, and the factors are named.

The second layer is the input layer, which is equivalent to a transfer station. It connects the processed data with the competitive layer and is responsible for transmission.

The third layer is the competition layer. The normalized data find the winning neuron by calculating the distance between the weight vector and the input vector of the mapping layer, update the weight of the adjacent neuron, and output the result after judging that it meets the conditions.

## 4. Empirical Analysis

The data in this paper come from the academic administration system of a certain college in a certain university to obtain the four-year academic performance information tables of 130 students of a certain major in 2016.

### 4.1. Factor Analysis Data Processing

First, the data of students' specific course records in the grade information table are cleaned. After data processing, the practical courses are combined into practical courses, and the common professional basic courses, professional core courses, and public compulsory courses are selected. Second, eliminate elective courses, screen and modify course name errors, remove missing exams, registration errors, and other noise data, and supplement a few missing grades with 60 points. The final data include variables such as student number, course name, and course score, and 37-course scores are obtained. According to the factor analysis theory, the experiment has 130 samples and 37 indicators, which *X*=(*X*_1_, *X*_2_,…,*X*_37_)^130^ are random vectors, and the common factor to be sought is *F*=(*F*_1_, *F*_2_,…,*F*_*m*_)^130^.

This section adopts the factor analysis method, and the software used is SPSS statistics 26.

First, the data are imported into the software for factor analysis. After standardizing the data, the KMO value is 0.879, greater than 0.5, and the significance level is significantly less than 0.05, indicating that the variables in this study are suitable for factor analysis. The output results are shown in Table 1.

Then, factor analysis was carried out on all variables to obtain the eigenvalues, variance contribution rate, and cumulative variance contribution rate of 37 variables. According to the research, the components with eigenvalues greater than 1 are selected as factors, and a total of 9 factors are extracted. As shown in Table 2, the cumulative contribution rate of the nine factors is 67.45%, more than 60%, which meets the requirements of factor analysis. The study can extract these nine factors.

The evaluation is based on the notice of the measures for the evaluation of students' comprehensive quality issued by a school, which is also the principle that this study should follow.

From the study of the component matrix of factor analysis, it is found that the common factors displayed by the component matrix are not obvious, and the interpretation of the common factors is slightly difficult. Therefore, in this study, the maximum variance method is used to rotate the component matrix and sort it by size to obtain the rotated component matrix.

Through the total variance interpretation after rotation, 9 factors are obtained, respectively, *F*_1_, *F*_2_,…, *F*_8_, *F*_9_. The factors are then named by the rotated matrix list of components. Sort the variables contained in each factor, find out the variables with larger data in the matrix table, observe the commonness between variables, and then get the name of each factor. The resulting factor naming table is shown in Table 3.

### 4.2. SOM Neural Network Model Analysis

This paper uses MATLAB software to input the obtained data into the software for operation [16].

**Figure 2:**
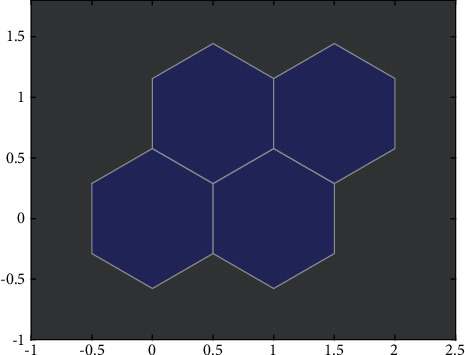
Hexagonal topology.

It can be seen from the input samples that the number of input neurons is 37. This study uses the hexagonal topology output. In the establishment of output layer neurons, there is no authoritative and effective theoretical method, so the trial-and-error method is used to establish the output layer neurons. Through many attempts, the number of output layer neurons is determined as 4, and the two-dimensional 2 × 2 SOM competition layer neurons are used as the capacity of clustering. The hexagonal topology is shown in Figure 2.

**Figure 3:**
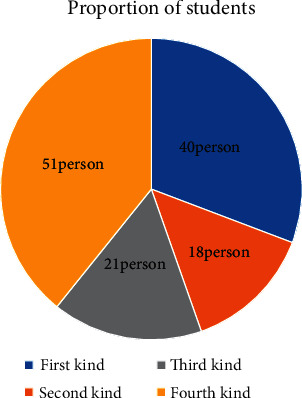
Classification results.

In the confirmation of training times, we can determine from the stability of the classification of training times. In this paper, the data are trained for 10, 25, 50, 100, 200, 500, and 1000 times, respectively, and the classification results after training are obtained. When the training times are 100 times, the classification results have been stable. Therefore, the training frequency of the study is 100 times. The training classification results are shown in Figure 3.

In other initial parameters, the default value of the topology function is “hextop,” and the default value of the distance function is “linkdish.” After all structures and initial parameters are established, the data are substituted into SOM network training. SOM network automatically looks for the nearest output neuron, finds the winning neuron, and records it. After reaching the training times, SOM clustering training is completed as shown in Table 4.

Through SOM neural network analysis, student groups can be divided into four categories. In order to more intuitively observe the proportion of students in each category, a pie chart of the proportion of students is drawn. At this time, we only get the number of people in each category, but the characteristics of these four categories are not known at present, so we will focus on exploring the characteristics of the four groups of people for analysis. The number and proportion of each category are shown in Figure 3.

Through the results of factor analysis in the previous article, the scores of students in each subject and the load after rotation are calculated, and the results are standardized to obtain the nine-dimensional comprehensive quality score of each student. Then, according to the analysis results of the SOM neural network, the students are divided into four categories, and the average value of nine-dimensional comprehensive quality indexes of each category of students is calculated [17, 18]. The statistical data obtained are shown in Table 5.

**Figure 4:**
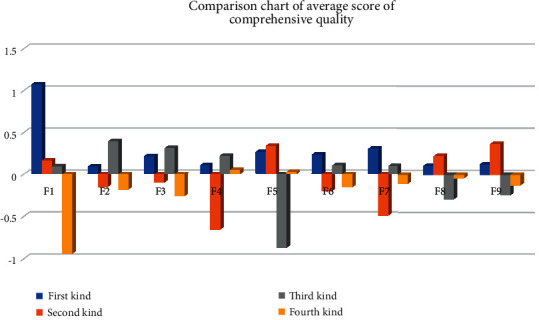
Comparison chart of average score of comprehensive quality.

In order to more intuitively observe the characteristics of each type of student, the average value of the comprehensive quality of the four types of students in Table 4 is converted into a bar chart. The abscissa represents each type of comprehensive quality, the ordinate represents the score of comprehensive quality, and different colors represent each type of student group.  Figure 4 shows the results.

The data in the table have been standardized, and the average value of each comprehensive quality is 0. Therefore, it can be seen from the above table and figure.

Compared with the top 40 students in this category, all of them have outstanding abilities.

There are 18 students in the second category. These students have obvious deficiencies in innovation and entrepreneurship ability, computer ability, physical quality, and language expression, but their professional core competence is relatively good.

There are 21 students in the third category. Their physical quality and mental health are relatively weak, and their scores in other aspects are higher than those in other categories, except for mathematical logical thinking ability. It can be seen that this kind of student's professional core ability is not strong.

There are 51 students in the fourth category, which is also the largest category. In addition to physical quality and mathematical logical thinking, the rest of these students are relatively low, indicating that they have obvious deficiencies and need to start from the foundation.

## 5. Conclusion

Through empirical analysis, the algorithm first classifies the students' comprehensive quality into nine categories based on the students' course scores by factor analysis, and the individual students can be evaluated by the classified data. Then, on this basis, SOM neural network clustering analysis is carried out, and students are divided into four categories. Students of different categories have corresponding characteristics, which can be evaluated for different student groups.

Aiming at the limitations of evaluating students' quality, the complexity of various data, and the evaluation based on the total score, this paper puts forward an improved SOM neural network model and adds factor analysis to the model. The model can not only extract the common factors in various disciplines, integrate various comprehensive abilities of students, but also improve the accuracy of clustering. The improved SOM model can evaluate the comprehensive quality of each type of student more intuitively and accurately and provide a strong basis for schools, teachers, and self-management, so as to promote the all-round development of students.

The improved SOM neural network algorithm is of great significance to the evaluation of students' comprehensive quality. The algorithm can reduce dimension and cluster data. However, when there are too many data dimensions, the operation difficulty of this model will increase, which also needs further improvement in the future. The algorithm can be applied in many aspects, not only to analyze students' comprehensive quality but also to evaluate and classify patients in hospitals. It is expected that the algorithm can be improved in the future, so as to make a more perfect evaluation of the comprehensive quality of students and evaluate the development of each student.

## Figures and Tables

**Table 1:** KMO and bartlett test.

KMO and bartlett test		
KMO sampling suitability quantity		0.879
Bartlett sphericity test	Approximate chi-square	2650.471
	Freedom	666
	Significance	0.000

**Table 2:** Interpretation of total variance after rotation.

Component	Initial eigenvalue			Sum of squares of rotating loads		
	Total	Percentage variance	Cumulative (%)	Total	Percentage variance	Cumulative (%)
1	12.371	33.434	33.434	9.281	25.085	25.085
2	2.623	7.089	40.523	2.451	6.625	31.709
3	2.353	6.360	46.883	2.447	6.614	38.323
4	1.571	4.245	51.128	2.269	6.132	44.455
5	1.516	4.096	55.224	1.952	5.276	49.731
6	1.277	3.451	58.675	1.950	5.271	55.002
7	1.190	3.217	61.892	1.780	4.812	59.814
8	1.049	2.836	64.728	1.429	3.861	63.675
9	1.010	2.729	67.457	1.399	3.782	67.457
10	……	……	……	……	……	……

**Table 3:** Factor naming table.

Component	Name	Factor naming
1	F1	Professional core competence factor
2	F2	Innovation and entrepreneurship ability factor
3	F3	Computer capability factor
4	F4	Physical literacy ability factor 1
5	F5	Logical thinking ability factor
6	F6	Political literacy ability factor
7	F7	Language expression ability factor
8	F8	Physical literacy ability factor 2
9	F9	Mental health ability factor

**Table 4:** Training classification results.

Number of training	1	2	3	4	5	6	7	8	9	10	11	12	13	14	15	16	17	18	…
10	2	4	1	3	1	3	2	3	2	2	1	1	3	1	4	1	3	3	…
25	3	1	4	2	4	2	3	2	3	3	4	4	2	4	1	4	2	3	…
50	3	4	1	2	1	2	3	2	3	3	1	1	2	1	4	1	2	2	…
100	3	4	1	2	1	2	3	2	3	3	1	1	2	1	4	1	2	2	…

**Table 5:** Average value of comprehensive quality of four types of students.

Name	Classification	One	Two	Three	Four
	Number of students	40	18	21	51
F1	Professional core competence	1.075113	0.1671	0.096766	−0.94205
F2	Innovation and entrepreneurship	0.096096	−0.15272	0.396673	−0.1848
F3	Computer capability	0.216969	−0.10328	0.313487	−0.2628
F4	Physical literacy ability 1	0.11106	−0.65692	0.223025	0.052913
F5	Mathematical logical thinking ability	0.269041	0.342395	−0.87165	0.027059
F6	Political literacy ability	0.237592	−0.20436	0.106884	−0.15823
F7	Language expression ability	0.305684	−0.493	0.104206	−0.10866
F8	Physical literacy ability 2	0.10712	0.222701	−0.29254	−0.04216
F9	Mental health ability	0.125481	0.366019	−0.24545	−0.12653

## Data Availability

All data, models, and code generated or used during the study appear in the submitted article.
